# Association between cardiac dysfunction and late gadolinium enhancement confined to the LV intramural region in patients with hypertrophic cardiomyopathy

**DOI:** 10.1080/07853890.2025.2533425

**Published:** 2025-07-23

**Authors:** Zhi Yang, Feng-you Yao, Yi-tian Long, Xue Meng, Liang-chao Gao, Yi You, Miao Wen, Shu-yue Pan

**Affiliations:** a Department of Radiology, Chengdu Fifth People’s Hospital, Chengdu, China; b Department of Cardiology, Chengdu Fifth People’s Hospital, Chengdu, China; c Department of Rheumatology and Immunology, Chengdu Fifth People’s Hospital, Chengdu, China

**Keywords:** Cardiac magnetic resonance imaging, late gadolinium enhancement, strain, hypertrophic cardiomyopathy

## Abstract

**Aim/Introduction:**

There are two major distributions of late gadolinium enhancement (LGE) in the context of hypertrophic cardiomyopathy (HCM): intramural LGE and LGE at right ventricular insertion points (RVIPs). However, the clinical significance of intramural LGE has not been well established.

**Materials and methods:**

A total of 117 consecutive patients with HCM (61 male; median age, 58.8 years) confirmed by cardiovascular magnetic resonance (CMR) were enrolled, and classified into three groups: (1) no LGE (*n* = 48), (2) intramural LGE (*n* = 49), and (3) RVIP LGE (*n* = 20).

**Results:**

Intramural LGE was detected in 41% of patients with HCM. HCM patients with intramural LGE had greater left ventricular (LV) wall thickness (LVWT) and greater LV mass than those without LGE (all *p* < 0.05). Furthermore, HCM patients with intramural LGE had a more depressed LV ejection fraction (LVEF) and more impaired global radial strain (GRS), global circumferential strain (GCS), and global longitudinal strain (GLS) than did those with RVIP LGE and those without LGE (all *p* < 0.05). Multivariate logistic regression analysis revealed that young age and severely thickened LVWT were associated with intramural LGE in patients with HCM (all *p* < 0.05). Furthermore, negative correlations were observed between intramural LGE and GRS, GCS, and GLS (all *p* < 0.001).

**Conclusions:**

Intramural LGE is associated with more severe HCM phenotypes, including a greater LVWT, LV mass and extent of LGE; a reduced LVEF; and impaired myocardial strain. These findings indicate that intramural LGE may be a noninvasive biomarker for risk stratification in patients with HCM.

## Introduction

Hypertrophic cardiomyopathy (HCM) is characterized by left ventricular (LV) hypertrophy in the absence of secondary causes and is the most common inherited cardiac disorder in young individuals [1]. Previous studies indicated that late gadolinium enhancement (LGE) detected by cardiovascular magnetic resonance (CMR) was present in 50 to 70% of patients with HCM [2]. However, several studies have confirmed that LGE is associated with adverse cardiovascular outcomes and a higher incidence of sudden cardiac death events in patients with HCM [3,4].

Significantly, the current guidelines state that the characteristics of LGE in patients with HCM reflect two major distributions of LGE: intramural LGE, which is LGE within the middle layer of the LV myocardium, and LGE at the anterior and/or posterior right ventricular insertion points (RVIPs) [5]. Interestingly, among these two LGE patterns, LGE involving only the RVIPs has not been shown to be associated with adverse cardiovascular outcomes in patients with HCM [6–9]. On the other hand, intramural LGE is thought to be an important LGE pattern that is confined to replacement fibrosis and may be a risk factor for adverse outcomes according to clinical work [10]. However, little has been reported on the effect of intramural LGE on LV structure and function using CMR in patients with HCM.

Therefore, this study aimed to characterize intramural LGE and to investigate the association between intramural LGE and LV function using CMR in HCM patients.

## Materials and methods

### Study population

This was a single-center retrospective observational cohort study. A total of 117 consecutive patients who were diagnosed with HCM with LGE and who underwent CMR between January 2021 and February 2023 were recruited. The diagnosis of HCM was confirmed using CMR imaging (LV wall thickness ≥ 15 mm or ≥ 13 mm with a family history of HCM). We excluded patients who had (1) a history of myocardial infarction or cardiac surgery, (2) a history of alcohol septal ablation, or (3) congenital heart disease or primary valvular heart disease. In addition, symptoms, blood test results, clinically relevant data, and N-terminal pro-brain natriuretic peptide (NT-proBNP) levels of eligible patients were recorded. This retrospective study was approved by the Ethics Committee of Chengdu Fifth People’s Hospital (approval number: 2024-072-01). Written informed consent for participation was obtained from all patients for inclusion in the study. In addition, the study was retrospective and all data were anonymised; therefore, the consent for publication was not required. The study was performed in accordance with the relevant guidelines and regulations and adhered to the principles of the Declaration of Helsinki.

### CMR image acquisition

Patients were scanned using a 3.0-Tesla MRI scanner (Vida, Siemens, Erlangen, Germany) with a 32-channel body coil. Both cine and LGE images in the 2- and 4-chamber long-axis LV views and the short-axis stack encompassing the ventricles (8 mm slice thickness and 2 mm gaps) were collected. In patients who underwent MRI, cine images were acquired using the balanced steady-state free-precession (SSFP) sequence (repetition time (TR): 39.12 ms, echo time (TE): 1.43 ms, flip angle (FA): 80°, field of view (FOV): 420 mm, matrix: 256 × 199, and phase: 25). LGE images were captured, on average, 10-20 min after injection of 0.1 mmol/kg gadobenate dimeglumine (MultiHance; Bracco) using the phase-sensitive inversion-recovery (PSIR) sequence (TR: 740 ms, TE: 1.06 ms, FA: 40°, FOV: 420 mm, and matrix: 256 × 144). The inversion time was optimized to nullify the normal myocardium.

### CMR analysis

We used CVI42 software (v. 5.15.4, Circle Cardiovascular Imaging, Calgary, Canada) for cine and LGE image analyses. The epicardial and endocardial borders of the LV myocardium were manually traced in all phases on short-axis cine images, and CVI42 software was used to calculate the following parameters: LV end-diastolic volume (LVEDV), LV end-systolic volume (LVESV), LV cardiac output (LVCO), LV cardiac index (LVCI), LV stroke volume (LVSV), LV ejection fraction (LVEF), right ventricular end-diastolic volume (RVEDV), right ventricular end-systolic volume (RVESV), right ventricular cardiac output (RVCO), right ventricular cardiac index (RVCI), right ventricular stroke volume (RVSV), right ventricular ejection fraction (RVEF), and LV mass. Papillary muscles were included in this volume. The LV mass-volume index was calculated as the LV mass divided by the LVEDV. LV wall thickness (LVWT) was measured in the end-diastolic phase of the short-axis cine images. CMR feature tracking (CMR-FT) strain measurements were obtained using the tissue feature tracking module in CVI42 software. Global radial strain (GRS), global circumferential strain (GCS), and global longitudinal strain (GLS) in the LV were assessed.

**Figure 1.**
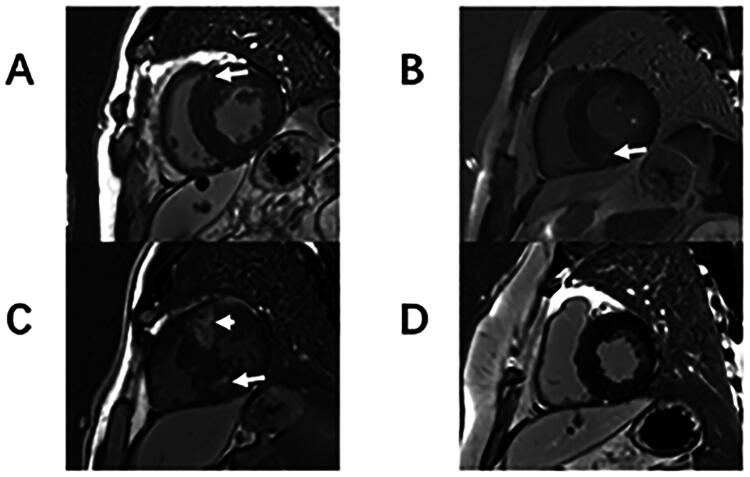
Late gadolinium enhancement (LGE) patterns for each group. Representative short-axis LGE images of HCM patients showing LGE located at right ventricle insertion points (RVIP LGE) only (B and D, white arrows) and patients with intramural LGE (C and D, yellow arrows). The figure in the bottom right corner shows a patient without LGE (A).

The presence and location of LGE were visually assessed by two observers with 3 years of CMR imaging experience. In cases of disagreement between the readers regarding the presence of LGE, a third reader assessed the images for final adjudication. The patients were then divided into three groups: (1) no LGE (no LGE) in the LV, (2) isolated LGE confined to the RVIPs (RVIP LGE), and (3) intramural LGE in the LV with or without RVIP LGE (Figure 1(A–D)). Patients without visible LGE in the LV were assigned a score of zero. The extent of LGE was defined using a threshold signal intensity of 5 standard deviations (SDs) from that of the normal myocardium and was calculated as the percentage of total LV mass (% LGE).

### Statistical analysis

Statistical analyses were performed using GraphPad Prism 7 (version 7.00, GraphPad Software, Inc.) or SPSS (version 23.0, released 2015, IBM Corp.), and we obtained a copyright license. Continuous variables are presented as the mean ± standard deviation (SD) or median and interquartile range (IQR), and categorical variables are presented as numbers with percentages. Comparisons among the three groups were made using ANOVA, and categorical variables were compared using the χ^2^ test, where appropriate. Univariate and multivariate logistic regression analyses were performed to assess predictors of intramural LGE. The associations between the variables in the subgroup were assessed using Spearman’s coefficient. A two-tailed *P* value less than 0.05 was considered significant.

## Results

### Baseline characteristics

This cohort included 129 consecutive patients with HCM. However, 12 patients were excluded due to a lack of LGE images or image artifacts, and 117 patients (52% male; median age, 58.8 years (50, 70)) with HCM were enrolled in total. Sixty HCM patients (51%) had hypertension, and 21 HCM patients (18%) had diabetes. Of the patients with HCM, 48 (41%) did not have LGE, and 69 (59%) had LGE. The baseline clinical characteristics of the patients with HCM are shown in Table 1.

**Table 1.** Baseline characteristics of HCM patients based on the presence of LGE on CMR.

	HCM with no-LGE (Group 1, *n* = 48)	HCM with Intramural–LGE (Group 2, *n* = 49)	HCM with RVIP-LGE (Group 3, *n* = 20)	ANOVA (P-value)
**Baseline characteristics**				
Age, years	64 (27.00, 70.00)	**53.18 ± 17.07** *	61.95 ± 14.59	0.002
Males, n (%)	21 (43.75%)	31 (52.54%)	9 (45.00%)	0.635
Height, cm	160 ± 7.71	162.4 ± 7.35	158.5 ± 9.07	0.171
Weight, Kg	61.3 (55.75, 70)	60 (55, 75)	61.71 ± 12.82	0.620
BMI, kg/m²	24.59 ± 3.29	24.42 (22.48, 27.33)	24.39 ± 3.68	0.921
Hypertension, n (%)	27 (60.00%)	23 (57.5%)	10 (50.00%)	0.941
Diabetes, n (%)	8 (18.18%)	9 (22.5%)	4 (22.22%)	0.871
Alcohol, n (%)	8 (18.18%)	16 (41.02%)	5 (27.78%)	0.071
Smoker, n (%)	12 (27.27%)	16 (41.02%)	3 (16.66%)	0.144
Systolic blood pressure, mmHg	122 (116.5, 131.5)	123 (116, 148.5)	126.1 ± 16.28	0.450
Diastolic blood pressure, mmHg	71.91 ± 12.13	74.5 (68.25, 84.75)	69.83 ± 8.40	0.176
Heart rate, bpm	72 (63.25, 81.5)	71 (64, 87.5)	71.1 ± 11.2	0.729
**Laboratory data**				
Hematocrit, %	41 (37.35, 45.05)	40.9 (36.93, 44.95)	40.5 (35.95, 43.1)	0.737
Blood glucose, mg/dL	5.94 (5.17, 7.85)	6.07 (5.32, 8.64)	6.24 (4.82, 7.99)	0.774
Hemoglobin A1c, mg/dL	6 (5.4, 6.85)	6.61 ± 1.41	6.43 ± 1.71	0.652
C-reactive protein, mg/dL	2.55 (0.9, 9.4)	2.1 (1.22, 3.77)	2.1 (1, 4.25)	0.780
NT-proBNP, pg/mL	874.4 (242.6, 1263)	**5799 ± 7107** *	1094 (266.9, 3981)	**0.035**
BUN, umol/L	7.53 ± 2.78	6.34 (5.32, 9.23)	7.53 ± 4.30	0.739
Serum creatinine, umol/L	69.3 (61.85, 79.65)	79.9 (66.25, 104.4)	71.25 (65.05, 95.48)	0.057
Total cholesterol, mmol/L	4.65 ± 0.93	4.63 (3.78, 5.35)	4.25 (3.88, 5.04)	0.750
Triglyceride, mmol/L	1.805 (1.20, 2.66)	1.86 (1.34, 3.05)	1.73 ± 0.97	0.256
HDL-cholesterol, mmol/L	1.19 (1.00, 1.48)	1.13 (0.96, 1.29)	1.24 ± 0.22	0.326
Troponin I, ng/mL	0.002 (0.001, 0.011)	0.027 (0.001, 0.279)	0.009 ± 0.005	0.057
Myoglobin, ng/mL	35.97 (27.37, 70.24)	58.19 (42.42, 78.9)	41.16 (33.3, 137.1)	0.078
CK-MB, U/L	10 (9, 13.75)	15 (10.5, 16.75)	13 (9.5, 14)	0.140

HCM, Hypertrophic Cardiomyopathy; Intramural-LGE, Late gadolinium enhancement present in middle layer of LV myocardium; RVIP-LGE, Late gadolinium enhancement present at the anterior and/or posterior right ventricular insertion points; CMR, Cardiovascular magnetic resonance; BMI, Body mass index; NT-proBNP, N-terminal prohormone of the brain natriuretic peptide; BUN, Urea nitrogen; HDL, High-density lipoprotein; CK-MB, Creatine kinase-MB; SD, Standard deviation; ANOVA, Analysis of Variance.

Continuous variables are reported as mean ± SD.

Categorical variables are reported as number (%).

Discontinuous variables are reported as median (25th-75th percentile).

Numbers in boldface indicate P values <0.05.

* *p* < 0.05 vs. without-LGE.

### Presence of intramural LGE

In the LGE group, 49 (72%) had intramural LGE, and 20 (28%) had RVIP LGE alone. In the intramural LGE group, 16 (33%) HCM patients had intramural LGE and RVIP LGE. Compared with HCM patients without LGE, HCM patients with intramural LGE were younger and more likely to have higher N-terminal prohormone of brain natriuretic peptide (NT-proBNP) levels (all *p* < 0.05). However, there were no differences between the intramural LGE and RVIP LGE groups in terms of age, sex, or NT-proBNP levels (all *p* > 0.05) (Table 1). In addition, patient comorbidities, such as diabetes, hypertension, alcohol consumption, and smoking, were similar among the no LGE, intramural LGE, and RVIP LGE groups (all *p* > 0.05).

A comparison of the CMR features of the three groups (Table 2) revealed that HCM patients with intramural LGE had a lower LVEF than those with RVIP LGE and those without LGE (all *p* < 0.05). Additionally, patients with intramural LGE had greater LVEDV and LVESV and greater maximum LVWT and LV mass than patients with HCM without LGE (all *p* < 0.05). However, no significant differences were observed in the maximum LVWT, LV mass, or LV mass-volume index between HCM patients with RVIP LGE and HCM patients without LGE (all *p* > 0.05). In addition, RV function, reflected by RVEF, RVEDV, RVESV, and RVSV, did not differ among the three groups (all *p* > 0.05). Similarly, patient comorbidities, such as LV obstruction, were not significantly different among the three groups (all *p* > 0.05).

**Table 2.** CMR Features of HCM patients depending on the presence and location of LGE on CMR.

	HCM with no-LGE (Group 1, n = 48)	HCM with Intramural-LGE (Group 2, n = 49)	HCM with RVIP-LGE (Group 3, n = 20)	ANOVA (*P*-value)	P 1 vs. 2	P 1 vs. 3	P 2 vs. 3
CMR findings							
LVEF, %	73.15 ± 10.8	57.94 ± 16.39	67.27 ± 9.56	**<0.001**	**<0.001**	0.223	**0.025**
LVEDV, mL	93.77 (70.81, 115.3)	110.4 (88.69, 138.7)	109.4 ± 27.82	**0.031**	**0.034**	0.290	>0.999
LVESV, mL	23.07 (15.87, 32.22)	36.75 (23.25, 63.53)	28.59(26.07, 46.65)	**<0.001**	**<0.001**	0.057	0.783
LVSV, mL	65.03 (51.78, 83.01)	65.8 ± 25.02	68.46 (55.15, 91.33)	0.392	>0.999	>0.999	0.519
LV mass, g	94.24 (77.61, 122.4)	140.7 (89.74, 167.2)	120.1 ± 35.67	**0.001**	**<0.001**	0.228	0.952
LV mass-volume index, g/ml	1.08 (0.90, 1.24)	1.16 (0.92, 1.41)	1.11 ± 0.24	0.394	0.549	>0.999	>0.999
LVWT, mm	16.00 (15.00, 17.00)	18.00 (16.00, 21.00)	18.4 ± 3.25	**0.003**	**0.004**	0.066	>0.999
RVEF, %	58.94 ± 12.23	51.7 ± 15.79	56.27 ± 13.79	0.079	0.074	>0.999	>0.999
RVEDV, mL	89.12 ± 27.48	91.36 (63.48, 117.1)	94.35 ± 32.7	0.841	>0.999	>0.999	>0.999
RVESV, mL	36.99 ± 16.79	38.83 (29.23, 51.9)	35.14 (27.18, 45.88)	0.696	>0.999	>0.999	>0.999
RVSV, mL	52.13 ± 17.46	48.97 ± 22.37	52.93 ± 22.71	0.395	0.729	0.988	0.755
LV obstruction, n (%)	16 (33.33%)	12 (24.48%)	7 (35.00%)	0.548	–	–	–
LGE extent, %	0	10.35 (6.70, 14.13)	5.8 (4.87, 7.70)	**<0.001**	**<0.001**	**<0.001**	**0.022**

HCM, Hypertrophic cardiomyopathy; LGE, Late gadolinium enhancement; CMR, cardiovascular magnetic resonance; LV, Left ventricular; LVEF, Left ventricular ejection fraction; LVEDV, Left ventricular end-diastolic volume; LVESV, Left ventricular end-systolic volume; LVSV, Left ventricular stroke volume; RVEF, Right ventricular ejection fraction, RVEDV, Right ventricular end-diastolic volume; RVESV, Right ventricular end-systolic volume; RVSV, Right ventricular stroke volume; LVWT, Left ventricular wall thickness; ANOVA, Analysis of Variance.

Continuous variables are reported as mean ± SD.

Categorical variables are reported as number (%).

Discontinuous variables are reported as median (25th-75th percentile).

Numbers in boldface indicate P values <0.05.

Compared with HCM patients with RVIP LGE alone, HCM patients with intramural LGE had a greater extent of LGE (*p* < 0.05).

### Global strain values in HCM patients with intramural LGE

**Figure 2.**
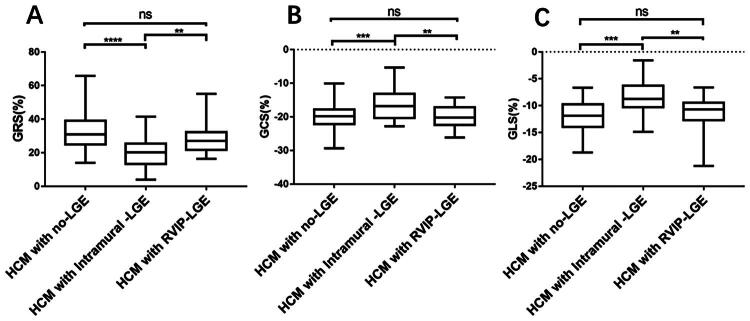
Differences in global radial strain (A, %), global circumferential strain (B, %) and global longitudinal strain (C, %) in HCM patients without late gadolinium enhancement (LGE), those with intramural LGE and those with LGE located in the right ventricle insertion points (RVIP LGE) only. **p < 0.01, ***p < 0.001, ****p < 0.0001.

Notably, the GRS, GCS, and GLS in the intramural LGE group were significantly lower than those in the RVIP LGE and no LGE groups (all *p* < 0.05) (Figure 2(A–C)). However, in HCM patients, the GRS, GCS, and GLS were not significantly different between patients with and without RVIP LGE (all *p* > 0.05).

### Predictive factors for intramural LGE

Univariate logistic analysis indicated that HCM patients who were younger than 60 years, male, had a maximum LVWT ≥ 19 mm, or had an LV mass-volume index ≥ 1.2 were more likely to present with intramural LGE (all *p* < 0.05) (Table 3). In addition, multivariate logistic regression analysis revealed that an age at illness onset of less than 60 years and severely thickened LVWT were associated with intramural LGE in HCM patients (all *p* < 0.05). However, we did not observe that sex, BMI, or the LV mass-volume index were associated with a high risk of intramural LGE according to multivariable logistic analysis (*p* > 0.05).

**Table 3.** Uni- and multivariable regression models for intramural LGE in patients with HCM.

Parameter	Univariable regression models		Multivariable regression models	
	OR (95%CI)	*P*-Value	OR (95%CI)	*P*-Value
Age < 60 years	3.1 (1.5-6.4)	**0.002**	2.6 (1.2-5.5)	**0.015**
Male	2.2 (1.1-4.5)	**0.027**	1.5 (0.7-3.3)	0.283
BMI ≥ 24	1.0 (0.5-2.1)	0.970	–	–
Max LVWT ≥ 19	3.8 (1.6-8.8)	**0.003**	2.7 (1.0-7.0)	**0.043**
LV mass-volume index ≥ 1.2	2.4 (1.1-5.3)	**0.022**	1.5 (0.6-3.6)	0.341

HCM, Hypertrophic Cardiomyopathy; Intramural-LGE, Late gadolinium enhancement present in middle layer of LV myocardium; RVIP-LGE, Late gadolinium enhancement present at the anterior and/or posterior right ventricular insertion points; OR, Odds ratio; CI, Confidence interval; BMI, Body Mass Index; LVWT, Left ventricular wall thickness; LVEF, Left ventricular ejection fraction; LV, Left ventricular; GLS, Global longitudinal strain.

Numbers in boldface indicate P values <0.05.

### Effect of intramural LGE on LV mechanics and remodeling in HCM patients

Significant negative correlations were demonstrated to exist between intramural LGE and GRS (R2=-0.4; *p* < 0.001), GCS (R2=-0.4; *p* < 0.001), and GLS (R2=-0.4; *p* < 0.001) in the HCM with LGE subgroup. There were similar correlations between intramural LGE and LVEF (R2=-0.3; *p* < 0.001) and age (R2=-0.3; *p* = 0.001). A positive correlation was found between intramedullary LGE and LV mass (R2 = 0.2; *p* = 0.035). However, intramural LGE was not associated with LVEDV, LV mass-volume index, BMI, or maximum LVWT in the HCM with LGE subgroup (all *p* < 0.05).

## Discussion

In our study, we showed that intramural LGE was present in approximately half of patients with HCM, and those with intramural LGE had a larger LV cavity size, greater LVWT/LV mass, and worse LV systolic function, which contributed to a reduced LVEF. Moreover, intramural LGE was associated with a young age at illness onset and an increased LVWT in patients with HCM. Therefore, intramural LGE in HCM patients may exacerbate the HCM phenotype in clinical practice.

In this study, intramural LGE was found in approximately 41% of HCM patients, but these patients were younger than those without LGE. Consistent with the findings of previous studies, our study confirmed that the prevalence of LGE is higher in young patients with HCM than in elderly patients with HCM [11]. This has been explained by the fact that LV remodeling is common in young HCM patients with severe LV hypertrophy and contributes to the high incidence of sudden death [12,13]. Moreover, NT-proBNP levels were elevated in HCM patients with intramural LGE compared with those without LGE. This finding suggests that intramural LGE may have a negative effect on dysfunction in patients with HCM.

In our cohort, HCM patients with intramural LGE had a greater maximum LVWT than did HCM patients without LGE. Wall thickening may occur in the anterior wall of the LV and is accompanied by increases in both LVEDV and LVESV, which contributes to LGE in patients with HCM [14,15]. In addition, although the LV mass in the HCM with intramural LGE group was significantly higher than that in the HCM without LGE group, the LV mass-volume indices were similar between the two groups. Similarly, our data suggest that intramural LGE in HCM patients may affect LV systolic function, leading to LVEF worsening [16]. Overall, HCM patients who have intramural LGE may be in the progression stage and have more risk factors, such as a greater maximum LVWT and greater LV mass.

However, compared with HCM patients without LGE, HCM patients with RVIP LGE showed no impairment in LVEF, indicating that their LV systolic function was unaffected by RVIP LGE. In line with a previous study, the LVEF in patients with intramural LGE was different from that in patients without LGE and RVIP LGE alone [7]. Moreover, another study confirmed that the extent of RVIP LGE was associated with LV diastolic dysfunction in HCM patients [17]. According to our study, HCM patients with RVIP LGE do not appear to have cardiac systolic dysfunction and may be in the intermediate stage between intramural LGE and no LGE.

A previous study revealed that myocardial strain decreased with LGE and was associated with myocardial hypertrophy in patients with HCM [18]. However, studies on the impact of intramural LGE on LV strain have long been neglected. In this paper, the global strain of HCM patients with intramural LGE was lower than that of HCM patients with isolated RVIP LGE and HCM patients without LGE. Moreover, intramural LGE correlated with impaired LV strain (GRS, GCS, and GLS), suggesting a mechanistic link between focal myocardial fibrosis and impaired systolic function in HCM patients [19]. Interestingly, we observed that the LV strain in HCM patients with RVIP LGE alone did not differ significantly from the LV strain in HCM patients without LGE. Therefore, our findings suggest that intramural LGE and RVIP LGE have different effects on LV morphology and function.

Although the mechanisms underlying the different patterns of LGE in patients with HCM remain to be fully elucidated, the following mechanisms may be relevant. Given the typical pattern and focused distribution of intramural LGE, replacement fibrosis is highly probable and was verified in a prior pathological study [20]. In addition, several studies have demonstrated that RVIP LGE may represent myocardial disarray rather than replacement fibrosis [20–22]. Presumably, intramural LGE in patients with HCM may result from an increase in replacement fibrosis *via* a different mechanism than RVIP LGE. Moreover, RVIP LGE is common even in healthy patients but does not convey a worse prognosis [23]. On the other hand, we also observed that the extent of LGE increased substantially more in HCM patients with intramural LGE than in those with RVIP LGE. Therefore, in our study, the differences in LV systolic function between the groups appeared to be driven by the differences in LGE and the extent of LGE. Specifically, patients with intramural LGE could have a more severe risk profile than those with RVIP LGE alone.

By using multivariate analysis, we focused on potential mechanisms underlying the development of intramural LGE in patients with HCM. In the present study, age and the maximum LVWT were predictive of intramural LGE in patients with HCM according to multivariate regression analysis. HCM patients with a high LV mass-volume index usually have a greater LV wall thickness. These patients tend to have intramural LGE and to experience disease onset at a younger age [12,24]. Additionally, myocardial intramural LGE was associated with decreased global strain values and was strongly predictive of worse outcomes [16]. Overall, intramural LGE can not only serve as a noninvasive screening marker but also benefit risk stratification in patients with HCM.

Our study had several limitations. First, the results were obtained from a single-center retrospective cohort of patients with HCM. Although we enrolled consecutive patients with HCM, selection bias may be present. Therefore, a larger prospective study is warranted. Second, all HCM patients included in our study met the criteria for a clinical diagnosis of HCM. However, only a small number of patients had undergone genetic diagnosis because genetic diagnosis is too expensive. Third, T1 mapping is a promising CMR technique for detecting diffuse myocardial fibrosis. However, T1 mapping, which could provide a potential biomarker for risk prognostication, was not performed in our study, and this will need to be addressed in future studies.

## Conclusion

In summary, our data indicate that intramural LGE is a readily observable, noninvasive biomarker associated with more advanced HCM, as reflected by its association with greater LV mass and LGE extent, reduced LVEF, and impaired myocardial strain. Patients with HCM and intramural LGE are more likely to have adverse clinical manifestations. These findings highlight the importance of intramural LGE in the risk stratification of patients with HCM, and future larger multicenter follow-up studies are needed to confirm this finding.

## Data Availability

The datasets used and/or analyzed during the current study are available from the corresponding author upon reasonable request.
